# Evaluation of Cardiovascular Risk Factors and Their Association With Coronary Artery Disease in Pakistani Adults

**DOI:** 10.7759/cureus.64670

**Published:** 2024-07-16

**Authors:** Muhammad Shah Nawaz Khan, Muhammad Imran Khan, Ali Saqlain, Shehryar Umer, Maithem Haider, Khubaib Ashiq

**Affiliations:** 1 Physiology, HBS Medical and Dental College, Islamabad, PAK; 2 Emergency Medicine, Shifa International Hospital, Islamabad, PAK; 3 Internal Medicine, Pakistan Air Force (PAF) Hospital, Islamabad, PAK; 4 Pathology, HBS Medical and Dental College, Islamabad, PAK; 5 Emergency Medicine, Rawalpindi Medical University, Rawalpindi, PAK

**Keywords:** cardiovascular diseases, traditional risk factors, pakistani adults, coronary artery disease (cad), cardiovascular risk factors

## Abstract

Background: Cardiovascular diseases (CVDs), especially coronary artery disease (CAD), are a major health burden, and their incidence is rising in countries like Pakistan.

Objective: The objective of this research was to assess the prevalence and association of cardiovascular risk factors with CAD in Pakistani adults.

Methodology: The research was carried out from January 2023 to December 2023 at the Pakistan Institute of Medical Sciences (PIMS) Islamabad and Shifa International Hospital Islamabad, Pakistan, using a cross-sectional design. Based on predefined criteria, 320 individuals who were Pakistani nationals and over the age of 18 years old were included. Those having a history of congenital heart disease, pregnancy, significant comorbidities, coronary artery bypass grafting (CABG), or percutaneous coronary intervention (PCI) were excluded. Medical, lifestyle, and demographic data were collected, and clinical evaluations were carried out by qualified healthcare workers. The data was analyzed using descriptive statistics and relevant statistical tests. A p<0.05 was statistically significant.

Results: The study investigated cardiovascular risk factors and demographic traits in 320 adult Pakistanis. The majority of participants (n=181; 56.56%) were male and aged between 18 and 45. University education was predominant (n=170; 53.13%). Specifically, 147 participants (45.94%) had dyslipidemia, 74 (23.12%) had diabetes mellitus, and 112 (35.0%) had hypertension. Diabetes mellitus (OR: 9.60, 95% CI: 4.52-20.38, p<0.001), dyslipidemia (OR: 1.88, 95% CI: 1.29-2.75, p=0.001), and hypertension (OR: 2.67, 95% CI: 1.72-4.15, p<0.001) showed significant correlations with CAD. Poor socioeconomic status (OR: 3.00, 95% CI: 1.31-6.89, p=0.009) and genetic propensity (OR: 2.00, 95% CI: 1.02-3.92, p=0.040) were also significantly associated with CAD.

Conclusion: Our study highlights diabetes as strongly linked to CAD in Pakistani adults, while socioeconomic status emerges as a significant predictor.

## Introduction

Cardiovascular diseases (CVDs) are a major source of morbidity and death, with coronary artery disease (CAD) being one of the main causes [1,2]. Even though our knowledge of the pathogenesis and treatment of CAD has advanced significantly, this condition still poses a serious threat to public health, especially in places like Pakistan [3]. An extensive examination of the risk factors for CAD and how they interact in Pakistani adults is necessary due to the alarming increased trend in the disease's incidence in this community [4].

Numerous modifiable and non-modifiable risk factors contribute to the development of CAD, making its etiology complex [5,6]. Conventional risk factors for CAD have been well-researched around the globe [7], including smoking, obesity, diabetes mellitus, dyslipidemia, and hypertension. However, owing to genetic, environmental, and behavioral variables, the incidence and effect of these risk factors may fluctuate across other populations [8]. Comprehending the distinct profile of cardiovascular risk factors in adult Pakistanis is crucial in order to formulate efficacious preventative measures and customized therapies [9].

Furthermore, new research indicates that the pathophysiology of CAD may include unique risk factors as genetic predisposition, inflammatory indicators, and socioeconomic determinants [10,11]. Recent research has emphasized the significant prognostic role of various biomarkers. Sharma et al. [12] demonstrated the efficacy of NLR as a cost-effective prognostic indicator in ST-elevation myocardial infarction (STEMI) [12]. Birdal [13] explored the association between the C-reactive protein-to-albumin ratio and long-term mortality in patients with acute coronary syndrome (ACS) [13]. Makkar et al. [14] investigated how fibrinogen, albumin, and the fibrinogen-to-albumin ratio correlate with angiographic severity and outcomes in ACS patients [14]. Additionally, Batta et al. [15] evaluated the impact of hs-CRP levels on outcomes in individuals with nonvalvular atrial fibrillation [15]. Integrating these findings with traditional risk factors such as genetic predisposition and socioeconomic determinants provides a more comprehensive understanding of CAD pathophysiology. This holistic approach not only strengthens risk assessment models but also supports the development of personalized prognostic and therapeutic strategies. Together with conventional risk variables, examining these components' roles may provide thorough insights into the illness process and help improve risk classification models for improved prognosis and treatment [16].

The amount of research on cardiovascular risk factors is increasing, but there is still much to learn about their particular significance and interactions with CAD in adult Pakistanis. Few research have thoroughly evaluated the frequency and significance of both established and emerging risk variables in this group. In order to create focused preventative and therapeutic strategies and lessen the prevalence of CAD in Pakistan, it is essential to address this gap. Therefore, in order to shed light on both existing and new causes of the illness, the purpose of this research is to assess the prevalence and correlation of cardiovascular risk factors with CAD in adult Pakistanis.

Research objective

The objective of this research was to assess the prevalence and association of cardiovascular risk factors with CAD in Pakistani adults.

## Materials and methods

Study design and settings

This research was conducted at the Pakistan Institute of Medical Sciences (PIMS) and Shifa International Hospital in Islamabad, Pakistan, utilizing a cross-sectional design. PIMS, one of the largest tertiary care facilities in the nation, provides comprehensive medical treatment to a diverse patient population. The study was conducted over a one-year period from January 2023 to December 2023.

Inclusion and exclusion criteria

Participants included in the study were required to be at least 18 years old, Pakistani nationals, and willing to participate voluntarily. Exclusion criteria encompassed individuals with a history of coronary artery bypass grafting (CABG) or percutaneous coronary intervention (PCI), congenital heart disease, pregnancy, or significant comorbidities that could affect their ability to participate effectively.

Sample size

Sampling methods involved systematic recruitment from hospital wards and outpatient departments. A sample size of 320 participants was determined based on estimated prevalence rates of cardiovascular risk factors and CAD in the Pakistani adult population, with a 95% confidence level and a 5% margin of error.

Data collection

Data collection took place from January 2023 to December 2023. Participants meeting the inclusion criteria were recruited from hospital wards and outpatient departments. The study utilized a standardized questionnaire (see Appendices) to gather data on lifestyle variables, medical history, and demographics. Clinical examinations, including blood pressure measurements, anthropometric assessments, and laboratory tests such as lipid profiles and fasting blood sugar levels, were conducted by trained medical personnel.

Statistical analysis

The individuals' risk factor profiles and demographic traits were compiled using descriptive statistics. Categorical data were shown as frequencies and percentages, while continuous variables were shown as mean±standard deviation (SD) or median with interquartile range (IQR). Logistic regression analysis was one of the relevant statistical methods used to evaluate the relationship between cardiovascular risk variables and CAD. At p<0.05, statistical significance was established.

Ethical approval

This study was conducted at two tertiary care hospitals in Islamabad, Pakistan. These hospitals were Shifa International Hospital and PIMS. Approval was obtained from the Institutional Review Board and Ethics Committee of Shifa International Hospital (approval number: 028-22) and the Hospital Ethics Committee of PIMS (approval number: ECPIMS/22/13). This study was conducted according to the ethical principles of the Declaration of Helsinki. Every participant gave their informed permission before being included in the study, and the confidentiality of the information gathered was maintained at every stage of the investigation.

## Results

The demographic details of the 320 participants are shown in Table 1. In terms of age distribution, the bulk of participants (118 or 36.88%) and the sample (102 or 31.88%) were in the 18-30 and 31-45 age categories, respectively. The lesser numbers of participants were in the 46-60 age group (63 or 19.69%) and the over 60 age group (37 or 11.56%). When it came to gender, 139 individuals (43.44%) were female, and 181 (56.56%) were male. The participants' levels of education varied: 170 individuals (53.13%) had a university education, 123 participants (38.44%) had attended college, and 27 participants (8.44%) had finished high school. The data about marital status revealed that 196 participants, or 61.25%, were married. Following them were 93 participants, or 29.06%, who were single and 31 participants, or 9.69%, who were divorced. When it came to employment, 250 participants, or 78.13%, were in the workforce, while 70 participants, or 21.87%, were jobless. Geographically, a larger percentage of participants lived in urban regions (241 or 75.31%) than in rural areas (79 or 24.69%).

**Table 1:** Demographic characteristics of participants (n=320)

Characteristic	Category	Frequency (n)	Percentage (%)
Age (years)	18-30	118	36.88
	31-45	102	31.88
	46-60	63	19.69
	>60	37	11.56
Gender	Male	181	56.56
	Female	139	43.44
Education level	School	27	8.44
	College	123	38.44
	University	170	53.13
Marital status	Single	93	29.06
	Married	196	61.25
	Divorced	31	9.69
Occupation	Employed	250	78.13
	Unemployed	70	21.87
Geographic location	Urban	241	75.31
	Rural	79	24.69

The 320 individuals' conventional cardiovascular risk factor prevalence is shown in Table 2. Of the sample, 208 people (65.00%) did not have hypertension, whereas 112 persons (35.00%) had hypertension. Of the sample, 147 people (45.94%) had dyslipidemia, whereas 173 participants (54.06%) did not have dyslipidemia. Out of the sample, 246 people (76.88%) did not have diabetes mellitus, whereas 74 participants (23.12%) reported having the condition. In terms of smoking tendencies, 102 individuals (31.88%) were past smokers, 171 participants (53.43%) were non-smokers, and 47 participants (14.69%) were current smokers. Of the individuals, 96 (30.00%) had a BMI of 30 or above, indicating that they were obese, and 224 (70.00%) had a BMI of less than 30.

**Table 2:** Prevalence of traditional cardiovascular risk factors (n=320) *BMI: body mass index

Risk factor	Category	Frequency (n)	Percentage (%)
Hypertension	Yes	112	35.00
	No	208	65.00
Dyslipidemia	Yes	147	45.94
	No	173	54.06
Diabetes mellitus	Yes	74	23.12
	No	246	76.88
Smoking	Current smoker	47	14.69
	Former smoker	102	31.88
	Non-smoker	171	53.43
Obesity	BMI ≥30	96	30.00
	BMI <30	224	70.00

The 320 individuals' unique cardiovascular risk factor prevalence is shown in Table 3. Two hundred and eighteen people (68.12%) showed normal levels of inflammatory markers, while 102 people (31.88%) had increased levels. Of the subjects, 239 (74.69%) showed no genetic tendency, whereas 81 (25.31%) showed genetic predisposition. The participants' socioeconomic positions varied; 66 (20.63%) were classified as low, 144 (45.00%) as intermediate, and 110 (34.37%) as high. Furthermore, of the 280 individuals (87.50%), 40 participants (12.50%) identified additional variables that increased their risk of cardiovascular disease.

**Table 3:** Prevalence of novel cardiovascular risk factors (n=320)

Risk factor	Category	Frequency (n)	Prevalence (%)
Inflammatory markers	Elevated	102	31.88
	Normal	218	68.12
Genetic predisposition	Present	81	25.31
	Absent	239	74.69
Socioeconomic status	Low	66	20.63
	Middle	144	45.00
	High	110	34.37
Others	Yes	40	12.50
	No	280	87.50

The clinical features of the 320 patients are shown in Table 4. The average BMI was 28.3±4.6, which falls into the overweight category. The mean diastolic blood pressure was 80±6 mmHg, while the mean systolic blood pressure was 130±10 mmHg. The interquartile range (IQR) for fasting blood sugar levels was 95-125 mg/dL, with a median of 110 mg/dL. The IQR for total cholesterol was 180-220 mg/dL, with a median of 200 mg/dL. These clinical measures provide light on the patients' state of cardiovascular health.

**Table 4:** Clinical characteristics of participants (n=320) BMI: body mass index

Characteristic	Mean±SD or median (IQR)
BMI	28.3±4.6
Systolic blood pressure	130±10 mmHg
Diastolic blood pressure	80±6 mmHg
Fasting blood sugar	110 mg/dL (95-125)
Total cholesterol	200 mg/dL (180-220)

The correlation between conventional risk factors and CAD in the 320 individuals is shown in Table 5. There was a substantial increase in the chance of CAD among those with hypertension, as shown by the odds ratio of 2.67 (95% CI: 1.72-4.15) and a p-value of <0.001 for those with hypertension compared to 41 without CAD. Similarly, there was a substantial correlation between dyslipidemia and CAD, as seen by the 91 instances of CAD among persons with dyslipidemia and the 56 cases without it, providing an odds ratio of 1.88 (95% CI: 1.29-2.75) and a p-value of 0.001. With 60 occurrences among those with diabetes and 14 among those without, diabetes mellitus demonstrated a robust correlation with CAD, yielding a high odds ratio of 9.60 (95% CI: 4.52-20.38) and a p-value of <0.001. However, risk ratios of 1.75 (95% CI: 0.88-3.48) and a p-value of 0.105 for current smokers and 1.38 (95% CI: 0.86-2.21) and a p-value of 0.183 for those with a BMI ≥30 did not show statistically significant relationships with CAD.

**Table 5:** Association between traditional risk factors and CAD (n=320) P-value <0.05 was significant CAD: coronary artery disease

Risk factor	Category	CAD	No CAD	Odds ratio (95% CI)	P-value
Hypertension	Yes	81	41	2.67 (1.72-4.15)	<0.001
Dyslipidemia	Yes	91	56	1.88 (1.29-2.75)	0.001
Diabetes mellitus	Yes	60	14	9.60 (4.52-20.38)	<0.001
Smoking	Current	30	17	1.75 (0.88-3.48)	0.105
Obesity	BMI ≥30	57	39	1.38 (0.86-2.21)	0.183

Table 6 presents the correlation between new risk variables and CAD among 320 individuals. Compared to 42 individuals without CAD, 58 instances of CAD had higher levels of inflammatory markers, resulting in an odds ratio of 1.50 (95% CI: 0.86-2.61) and a p-value of 0.158, indicating a non-significant correlation between raised inflammatory markers and CAD. There was a substantial correlation between genetic predisposition and CAD, with an odds ratio of 2.00 (95% CI: 1.02-3.92) and a p-value of 0.040, comparing the 51 instances of CAD with the 30 cases without CAD that had genetic predisposition. There was also a statistically significant correlation between socioeconomic status and CAD, with 30 instances among those classified as low status and 36 cases among those without CAD, yielding an odds ratio of 3.00 (95% CI: 1.31-6.89) and a p-value of 0.009. Other covariates, however, did not demonstrate a statistically significant connection with CAD, as shown by an odds ratio of 1.00 (95% CI: 0.51-1.96) and a p-value of 0.999 for other risk factors.

**Table 6:** Association between novel risk factors and CAD (n=320) CAD: coronary artery disease

Risk factor	Category	CAD (n)	No CAD (n)	Odds ratio (95% CI)	P-value
Inflammatory markers	Elevated	58	42	1.50 (0.86-2.61)	0.158
Genetic predisposition	Present	51	30	2.00 (1.02-3.92)	0.040
Socioeconomic status	Low	30	36	3.00 (1.31-6.89)	0.009
Others	Yes	18	22	1.00 (0.51-1.96)	0.999

## Discussion

The results of this research highlight how important it is to have a thorough knowledge of cardiovascular risk factors and how they relate to adult CAD in Pakistan. Examining the frequency of conventional risk factors, our findings showed significant percentages of people with diabetes mellitus, dyslipidemia, and hypertension, which is in line with worldwide patterns [17]. The strong correlation between diabetes mellitus and CAD was especially noteworthy. People with diabetes had a significantly higher likelihood of developing CAD than people without the disease (CAD: 60 vs. no CAD: 14), yielding a high odds ratio of 9.60 (95% CI: 4.52-20.38) [18]. Moreover, recent studies by Otaal et al. [19] and Bouisset et al. [20] consistently demonstrate that diabetes mellitus is associated with worse overall outcomes and decreased viability among CAD patients, including those with ACS. This result emphasizes how crucial strict diabetes control is as a primary preventative measure against CAD in this group.

Moreover, whereas obesity and smoking are known risk factors for CAD worldwide, our research did not uncover any statistically significant correlations between these variables with CAD in adult Pakistanis [21-23]. Smoking, in particular, did not show statistically significant correlations with CAD; hazard ratios for current smokers were 1.75 (95% CI: 0.88-3.48). Likewise, there were no statistically significant correlations between obesity, defined as having a BMI of 30 or higher, and CAD, with odds ratios for those with a BMI of ≥30 being 1.38 (95% CI: 0.86-2.21). This interesting disparity may call for more research into possible hereditary or cultural variables affecting how these risk factors affect this community [24,25]. It also highlights the need for customized therapies that address the distinct risk factor profile seen in Pakistani people.

Our research investigated the involvement of new variables such as inflammatory markers, genetic predisposition, and socioeconomic status in the pathogenesis of CAD in addition to conventional risk factors. Socioeconomic status emerged as a significant predictor of CAD, with individuals from lower socioeconomic strata showing a threefold higher likelihood of CAD compared to their counterparts (low SES: 30 vs. no CAD: 36), resulting in an odds ratio of 3.00 (95% CI: 1.31-6.89) [26]. Elevated levels of inflammatory markers and genetic predisposition also showed varying degrees of association with CAD. This demonstrates the complex relationship that exists between socioeconomic inequalities and cardiovascular health outcomes, highlighting the need for focused interventions that target social determinants of health in order to lower the prevalence of CAD in communities who are already at risk [27].

The fact that our study fills a knowledge vacuum in cardiovascular risk factors unique to the Pakistani population is remarkable. Our results provide light on the frequency and correlation between established and new risk factors and CAD, which might be useful information for researchers, policymakers, and medical professionals as they develop evidence-based plans for the treatment and prevention of CAD in Pakistan. To confirm these results and clarify the underlying processes behind the observed relationships, further long-term research is necessary. One limitation of the study is its cross-sectional design, which provides a snapshot of data at a specific point in time. This type of study can establish associations between cardiovascular risk factors and CAD but cannot determine causality or account for changes over time. The findings may be influenced by selection bias, as the sample was drawn from specific hospitals in Islamabad, limiting generalizability to the broader Pakistani population. Additionally, cross-sectional studies may not capture the dynamic nature of CAD progression or the cumulative effects of risk factors. Future research employing longitudinal designs and a more diverse participant pool could provide deeper insights into the causal relationships and long-term impacts of these risk factors on CAD in Pakistani adults.

## Conclusions

The significance of comprehending cardiovascular risk variables and their correlation with CAD in adult Pakistanis is highlighted by our research. Despite the prevalence of conventional risk factors such as diabetes mellitus, dyslipidemia, and hypertension, we discovered a substantial correlation between diabetes and CAD. It's surprising that in this sample, obesity and smoking did not significantly correlate with CAD, pointing to the possibility of other factors at work. Furthermore, it was shown that socioeconomic position was a major predictor of CAD, emphasizing the need for focused treatments that address social determinants of health. These results provide valuable information for developing customized measures to reduce the prevalence of CAD in Pakistan; however, further investigation is necessary to validate these correlations and investigate underlying causes.

## Appendices

**Table 7:** Questionnaire on demographics, medical history, and cardiovascular risk factors BMI: body mass index

Section	Question	Options
Demographics	What is your age?	A: 18-30
		B: 31-45
		C: 46-60
		D: >60
	What is your highest level of education?	A: School
		B: College
		C: University
	What is your marital status?	A: Single
		B: Married
		C: Divorced
	What is your current employment status?	A: Employed
		B: Unemployed
	Where do you reside?	A: Urban
		B: Rural
Medical history	Do you have a history of hypertension?	Yes
		No
	Do you have a history of dyslipidemia?	Yes
		No
	Do you have a history of diabetes mellitus?	Yes
		No
	Are you currently a smoker?	A: Current smoker
		B: Former smoker
		C: Non-smoker
	Is your BMI ≥30?	Yes
		No
Novel risk factors	Do you have elevated inflammatory markers?	Yes
		No
	Do you have a genetic predisposition for cardiovascular diseases?	Yes
		No
	What is your socioeconomic status?	A: Low
		B: Middle
		C: High
	Are there any other factors that increase your risk of cardiovascular disease?	Yes
		No
Clinical measures	What is your BMI?	
	What is your systolic blood pressure?	
	What is your diastolic blood pressure?	
	What is your fasting blood sugar level?	
	What is your total cholesterol level?	
